# The Diagnosis and Management of Chronic Constipation in Italy: Results from a Survey Conducted among Italian Gastroenterologists

**DOI:** 10.3390/jcm13206047

**Published:** 2024-10-10

**Authors:** Christian Lambiase, Lucia D’Alba, Francesca Galeazzi, Gabrio Bassotti, Danilo Consalvo, Edda Battaglia, Giovanni Cataudella, Maria Cristina Neri, Claudio Londoni, Piera Rossitti, Emiliano Valenzi, Bruno Annibale, Marco Soncini, Maria Caterina Parodi, Massimo Bellini

**Affiliations:** 1 Gastrointestinal Unit, Department of Translational Research and New Technologies in Medicine and Surgery, University of Pisa, 56126 Pisa, Italy; 2 NIHR Nottingham BRC, Nottingham University Hospitals NHS Trust and the University of Nottingham, Nottingham NG7 2UH, UK; 3 Nottingham Digestive Diseases Centre, Translational Medical Sciences, School of Medicine, University of Nottingham, Nottingham NG7 2UH, UK; 4 Department of Gastroenterology and Endoscopy, San Camillo Forlanini Hospital, 00152 Rome, Italy; 5 Gastroenterology Unit, Azienda Ospedale Università di Padova, 35128 Padua, Italy; 6 Gastroenterology & Hepatology Section, Department of Medicine and Surgery, University of Perugia, 06156 Perugia, Italy; 7 Gastroenterology Unit, Perugia General Hospital, 06156 Perugia, Italy; 8 Department of Gastroenterology and Digestive Endoscopy, AORN “Antonio Cardarelli”, 80131 Napoli, Italy; 9 Gastroenterology Unit, ASL TO4 Chiavasso–Ciriè–Ivrea, 10034 Torino, Italy; 10 Gastroenterology and Endoscopy Outpatient Clinic, Casa di Cura Eretenia, 36100 Vicenza, Italy; 11 Gastroenterology Unit, Geriatric Institute Pio Albergo Trivulzio, 20146 Milan, Italy; 12 Gastroenterology and Endoscopy Department, Maggiore Hospital, 26013 Crema, Italy; 13 Ambulatorio Perineologico Riabilitativo, 33100 Udine, Italy; 14 Gastroenterology Unit, ASL Latina, 04100 Latina, Italy; 15 Department of Medical-Surgical Sciences and Translational Medicine, Sant’Andrea Hospital, Sapienza University of Rome, 00189 Rome, Italy; 16 Department of Medicine, ASST Lecco, 23900 Lecco, Italy; 17 Department of Gastroenterology and Digestive Endoscopy, San Martino Hospital, 16132 Genoa, Italy

**Keywords:** constipation, chronic constipation, disorders of gut–brain interaction, digital rectal examination, fiber, macrogol, anorectal physiology testing

## Abstract

**Background:** Chronic constipation (CC) is one of the most common disorders of gut–brain interaction (DGBI). The management of CC requires specific skills due to its complex and multifactorial pathophysiology and its multistep treatment. The aims of this study were to evaluate the availability and the use of diagnostic tools for CC in Italy and the therapeutic management of CC by Italian gastroenterologists (GEs). **Methods:** A survey was conducted during the 28th meeting of the Italian Federation of Digestive Disease Societies (FISMAD; Rome, Italy, 11–14 May 2022). The survey explored the presence of a clinic dedicated to DGBIs, the availability and the use of specific diagnostic tools, the routine use of digital rectal examination (DRE), and the therapeutic approach to CC by Italian GEs. **Results:** The survey was taken by 236 GEs. The most significant results were that 42% of respondents had a clinic dedicated to DGBI in their institute; DRE was regularly performed by 56.8% of GEs when evaluating a CC patient; young GEs (≤40 years) performed DRE less frequently than older ones (*p* < 0.001); anorectal manometry was available to 44.3% of GEs; balloon expulsion test (BET) was available to 19.1% of GEs; GEs with a clinic dedicated to DGBI had more frequent access to anorectal physiology testing (*p* < 0.001); diet and lifestyle advice were the most frequently prescribed treatments; and fiber and macrogol were the second and third most prescribed treatments, respectively. **Conclusions:** The survey provides an interesting picture of CC management by Italian GEs. The results are in line with previous data collected about 10 years ago among Italian GEs (“CHRO.CO.DI.T.E study”); DRE is still rarely performed by Italian GEs (particularly by young GEs). The availability of anorectal physiology testing is still limited, and BET, which could be easily performed in everyday clinical settings, is rarely performed. Lifestyle suggestions, macrogol and fiber are the preferred treatment, as recommended by all guidelines. These results will be useful to identify as yet unmet educational needs and critical issues to improve CC management.

## 1. Introduction

Chronic constipation (CC) is a common disorder of gut–brain interaction (DGBI), affecting about 15% of the general population [1,2]. However, the prevalence of CC is underestimated. In fact, only one third of constipated patients seek medical advice and very few of them consult a gastroenterologist (GE) [3]. Indeed, constipated patients usually self-manage their problem, often by means of over-the-counter products or food supplements [3,4]. Functional constipation (FC), the most common subtype of CC, is defined by the Rome IV criteria [5]. Patients affected by irritable bowel syndrome with predominant constipation (IBS-C) [5] or patients with a functional defecation disorder [6] represent other frequent subtypes of CC. Secondary constipation, as a consequence of drugs or of organic disease, is another major problem that should not be overlooked [7,8].

CC has a negative impact on one’s social, affective and professional life [9,10,11,12,13,14], and it is also associated with a reduction in a patient’s quality of life (QoL) and represents a heavy economic burden [9,10,11,12,13,15]. The latter also affects inpatients; indeed, the number of inpatient discharges for constipation has increased in recent decades [16]. Most epidemiological studies indicate a higher prevalence of constipation and laxative use in the elderly, particularly in those institutionalized, with a prevalence of up to 50%. About 74% of nursing home residents use daily laxatives [17,18,19].

This condition and its possible impact on QoL [20] tend to be underestimated by physicians. Furthermore, many physicians frequently consider reduced stool output as the only symptom related to constipation [21,22]. However, CC may include not only a reduced frequency of bowel movements but also hard stools, excessive straining to defecate, a sense of anorectal blockage, need for manual maneuvers, and a sense of incomplete evacuation after defecation [5].

The correct management of CC requires a complex, multidimensional approach. It is important to exclude secondary causes of CC, evaluate the presence of comorbidities and, in some instances (i.e., in refractory cases), explore the underlying pathophysiological mechanisms in order to improve management and therapeutic appropriateness [7,23,24]. In recent years, several surveys have been published regarding the management of CC [2,8,25,26,27,28]. These surveys were mainly directed at patients and addressed prevalence and risk factors [2,25,26] or treatment strategies [8,27,28] for patients with chronic constipation.

The aims of this survey were to evaluate the availability and use of diagnostic tools for CC in Italy and the therapeutic management of CC by Italian GEs.

## 2. Materials and Methods

During the 28th meeting of the Italian Federation of Digestive Disease Societies (FISMAD) (Rome, Italy, 11–14 May 2022), 236 GEs filled in a questionnaire about the ambulatory activities, diagnosis and management of patients suffering from CC.

The questionnaire was an example of simple data collection, with questions focused on the clinical management of constipation based on international guidelines [7] and, therefore, did not need validation.

### 2.1. Questionnaire

The survey consisted of questions with multiple choice answers (see the Supplementary Materials section), concerning the following:

- Demographic features of GEs (e.g., age, gender, geographical area of residence, working institution).
- The presence of a clinic dedicated to DGBI in the institution where they worked.
- The routine use of specific instruments to evaluate CC patients (e.g., Rome IV criteria [5], scores to assess both the severity and the impairment of quality of life induced by CC, Bristol Stool Scale).
- The routine use of digital rectal examination (DRE) when evaluating CC patients.
- The availability of the specific diagnostic tests (mainly anorectal physiology testing) used to evaluate patients in accordance with the international guidelines [7].
- The availability of a multidisciplinary approach by different specialists.
- The availability of pelvic floor rehabilitation professionals.
- The therapies usually prescribed by GEs to their CC patients.

The questionnaire was totally anonymous. The GEs had only to declare their consent to participate. This study was conducted according to the principles of the Helsinki Declaration, and it did not require the consent of any Ethics Committee.

### 2.2. Statistical Analysis

Descriptive statistics were used to define baseline demographic characteristics of GEs who took part in the survey. The Shapiro–Wilk test was used to assess the normality of the data. Data were provided as the median value (interquartile range, IQR). Quantitative data were analyzed by Mann–Whitney’s U test or/and the Kruskall–Wallis test when appropriate. An analysis of qualitative data was conducted using the Chi-square test. Data regarding therapies were analyzed by descriptive analysis. Regarding the therapies, each participant formulated a ranking on the prescription of eighteen treatments for constipation from the first to the eighteenth choice. For the first preferred therapy, it was possible to choose more than one option. Each therapeutic approach was presented as a frequency in each therapeutic preference. Data were tabled in Microsoft Excel 2010 (Microsoft Inc., Redmond, WA, USA) and analyzed with SPSS 28 (SPSS Inc., Chicago, IL, USA), and values of *p* < 0.05 were considered significant.

## 3. Results

### 3.1. Demographic Features

Two-hundred and thirty-six GEs took part in the survey and completed the questionnaire. The demographic features of the participants are reported in Table 1.

The median age of the respondents, equally represented by gender, was 48 years. Residents made up 12.9% of all respondents. Most respondents practiced in northern Italy (49.57%), followed by southern Italy and islands (33.47%) and central Italy (16.95%). There was no significant difference between gender among geographic areas. GEs from southern Italy and islands were significantly older compared with GEs from central Italy (median age 55 (IQR 20.3) vs. 44 (IQR 28.0) years, respectively; *p* = 0.016). Most respondents worked in non-university hospitals (52.7%); 30% worked in university hospitals, while the others worked in healthcare residences, territorial outpatient clinics and other healthcare structures. The average time to complete the survey was 10 min (range 8–15). No incentives were offered to GEs to complete the survey.

### 3.2. Use of Specific Scores and Questionnaires to Evaluate CC Patients

The survey addressed the utilization of specific questionnaires and scores while evaluating a CC patient. The participants were asked whether they regularly used the following questionnaires (Table 2): the Rome IV criteria questionnaires, scores to assess the severity of CC symptoms, scores to assess the impact of CC on quality of life (QoL) and the Bristol Stool Scale.

Systematic use of the Rome IV criteria and Bristol Stool Scale was reported by 75.0% and 74.1% of respondents, respectively, with no difference in gender, age, geographic area or type of healthcare structure. Conversely, symptom severity scores and QoL scores were infrequently used in clinical practice (38.6% and 36.4%, respectively). Younger GEs (≤40 years) used specific scales less frequently than older colleagues (>40 years) (for symptom severity 23.9% vs. 49.3% *p* < 0.01; for QoL 17.4% vs. 48.6% *p* < 0.01).

### 3.3. Digital Rectal Examination

Participants were asked whether DRE was routinely performed while evaluating a constipated patient. Slightly more than half of the total respondents (56.8%) reported that they always perform DRE when evaluating a constipated patient (Table 2). Notably, older GEs (>40 years) said they performed DRE more often compared to younger GEs (72.9% vs. 31.5%, respectively; *p* < 0.01).

### 3.4. Diagnostic Test Availability for CC

Data regarding the availability of diagnostic tests for CC are summarized in Table 3. Colonoscopy was available for most respondents (97.4%), while CT–colonoscopy was available for 73.3%. Transanal ultrasound and colonic transit time with radiopaque markers were available for 52.5% and 51.7% of respondents, respectively. ARM, BET and defeco-MRI had low rates of availability: conventional ARM was available for 41.1% of respondents, while high-resolution/high-definition ARM was available for 15.2%. Overall, 44.3% of GEs had access to at least one type of ARM. Defeco-MRI and BET were reported to be available for only 31.8% and 19.1% of GEs, respectively.

Significantly different distributions were found among the Italian geographic areas concerning CT–colonoscopy, conventional and high-resolution/high-definition ARM, balloon expulsion test (BET), transanal ultrasound, perineography and colonic transit time with radiopaque markers (Table 3).

### 3.5. DGBIs Outpatient Clinic Availability

Only 42% of GEs reported having a DGBI outpatient clinic in their working structures with no variation in distribution among Italian geographic areas. Interestingly, the presence of such an outpatient clinic positively influenced the more frequent use of scales and questionnaires to assess constipation. We go into more detail below (Table 2):

- Diagnosis through the Rome IV criteria was performed by 80.8% of those having a DGBI outpatient clinic vs. 70.8% of those not having a DGBI outpatient clinic (*p* < 0.05);
- Assessment of CC symptom severity by specific questionnaire was performed by 51.5% of those having a DGBI outpatient clinic vs. 30.7% of those not having a DGBI outpatient clinic (*p* < 0.01);
- Evaluation of CC impact on QoL by specific questionnaire was performed by 44.5% of those having a DGBI outpatient clinic vs. 30.7% of those without a DGBI outpatient clinic (*p* < 0.01);
- Assessment of fecal consistency through Bristol Stool Scale was performed by 88.0% of those having a DGBI outpatient clinic vs. 68.6% of those not having a DGBI outpatient clinic (*p* < 0.01).

**Figure 1:**
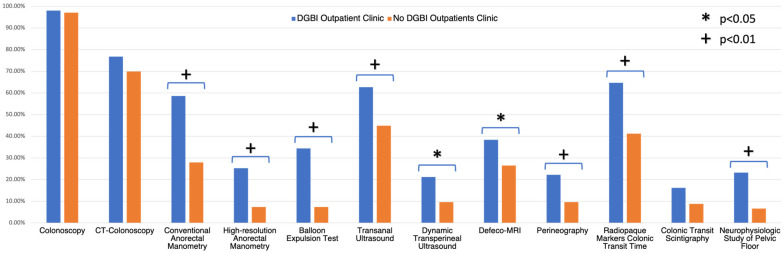
Diagnostic test availability according to presence or absence of a DGBI outpatient clinic.

Furthermore, having a DGBI clinic was associated with a more frequent routinely used multidisciplinary approach with other specialists; the most frequent collaborations were with surgeons (45.1%) and radiologists (36.3%). Moreover, specific diagnostic tests to assess constipation were more accessible to GEs having a DGBI outpatient clinic in their structure. ARM (both conventional and high-resolution/high-definition ARM), balloon expulsion test, transanal and transperineal ultrasound, defeco-MRI, perineography, colonic transit time with radiopaque markers and neurophysiologic study (Figure 1) were more frequently carried out in structures with a DGBI clinic.

### 3.6. Therapy for CC

**Figure 2:**
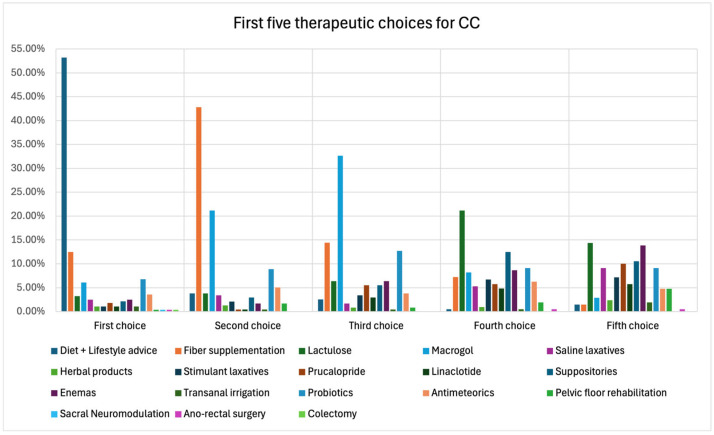
First five therapeutic choices for CC management.

GEs were asked to list 18 possible choices regarding CC treatments in order of preference from the most frequently chosen to the least frequently chosen. Diet and lifestyle advice were the preferred treatments as the first choice. Fiber supplementation was in second place, whereas macrogol came in third. Lactulose was the preferred fourth choice, while lactulose and enemas were the preferred therapeutical approaches most prescribed as the fifth choice. Suppositories were the second or third most prescribed therapeutical approaches in the fourth and fifth choice, respectively (Figure 2). Transanal irrigation (TAI) and pelvic floor rehabilitation (PFR) were mainly prescribed between the twelfth and fifteenth place. The presence of professionals dedicated to pelvic floor rehabilitation (PFR) and the type of PFR available in the structures was also assessed. Of the respondents, 44.5% had PFR available in their working structure, and the most available PFR technique was biofeedback. Having a DGBI clinic was associated with more frequent availability of a PFR clinic in its own structure (*p* < 0.01).

Finally, surgical and para-surgical approaches were the last therapeutic choices. The complete data regarding the therapeutic preference for each choice can be found in Figure S1 of the Supplementary Materials.

## 4. Discussion

Constipation ranks among the top five most common physician diagnoses for gastrointestinal disorders among outpatient clinic visits [29]. CC is a complex disease, which requires a multidisciplinary, time-consuming approach and specific skills [30]. The diagnostic workout of CC often includes ARM, balloon expulsion test, whole gut transit study and perineography [7]. All this necessary workout requires expertise and results in a heavy healthcare and economic burden [15].

We performed our survey to assess the point of view of Italian GEs in order to obtain a clear picture regarding the evaluation and management of CC in our country. Firstly, even if constipation is a very widespread condition, there are very few specialized outpatient clinics. Considering that FC and IBS-C are two of the most common subtypes of CC, it is notable that less than half of respondents had a clinic dedicated to DGBI in their institute (without any different distribution among Italian geographic areas). Effectively, this demonstrates that these disorders are somewhat underestimated in their importance by the National Health System and by GEs themselves. This leads to a paucity of referral centers, especially for the most complex patients. Multiple ineffective therapies, the continuous search for well-being, useless examinations and emergency department admissions for CC are associated with a high healthcare burden [15,16] and suggest that there is currently only partially effective management of this condition [31].

Proper treatment of DGBI is crucial and can lead to an improvement in economic burden. Maybe the fact that this condition does not put people’s lives at risk (while having a significant impact on quality of life [9,10,11,12,13,15]) leads to directing limited healthcare resources toward more “serious”, although less widespread, conditions. Our results suggest that having such a dedicated clinic in the structure could improve the management of constipated patients. GEs who had a DGBI clinic in their structure used scores more frequently to assess CC symptom severity or impact on QoL. In this regard, international guidelines point out the importance of taking a careful medical history of these patients [7], along with validated questionnaires and scores. This suggests that those having more knowledge of the topic may use the appropriate scores more often to give a standardized and objective evaluation of patients, as suggested by the guidelines [7]. Unfortunately, their routine use is limited by their scarce diffusion and by the time required for their administration to patients. Developing simpler or immediately validated tools to assess symptom severity and QoL might very well facilitate their introduction into everyday clinical practice.

Only 56.5% of GEs reported that they always perform DRE while evaluating a constipated patient. Younger GEs (≤40 years) perform it less frequently than older colleagues (Table 2, *p* < 0.001). Once again, our data are in line with previously published results and confirm that the overall routine use of DRE while evaluating a CC patient is relatively low among Italian GEs [32]. International guidelines and the recent literature strongly suggest always performing DRE while evaluating CC patients [7,23,33]. DRE can provide useful information about possible stool presence and consistency, anorectal masses, hemorrhoids, anal fissures, rectal prolapse or rectoceles. Moreover, it can guide diagnostic approaches and therapies because it provides useful information about anorectal function and coordination [34,35]. DRE should always be performed, together with anal relaxation and evaluating the abdominal contraction on straining, as together they may predict a functional defecation disorder [23]. An accurate DRE may also detect the early onset of anorectal cancer [30]. Given the importance of this diagnostic tool, why is DRE so infrequently used, especially by younger GEs? There is the possibility that various GEs find DRE unpleasant and feel embarrassed by performing it. Moreover, many patients are quite embarrassed about undergoing a DRE and are frequently fearful of experiencing discomfort. Furthermore, in some cases, GEs may avoid performing DRE for fear of medical–legal problems, especially where a nurse or a specializing doctor cannot be present. For these reasons, asking the patient to fill out an informed consent form before performing DRE could be important. Not performing DRE, especially on the part of young GEs, implies educational problems in specialized training courses. This suggests a pressing need to educate young GEs in the routine use of DRE starting from residency programs.

GEs who had a clinic dedicated to DGBI also had more frequent access to anorectal physiology testing (*p* < 0.001) (58.6% vs. 27.9% for ARM, 34.3% vs. 7.3% for BET) even though their overall availability was still limited. For example, BET, which can be performed easily in any clinical setting, is rarely used (by only about 19% of respondents). This is notwithstanding data in the literature showing the clinical relevance of this simple test, able to predict the biofeedback response (alone or combined with ARM) [36,37]. Therefore, some recent data suggest using BET in all clinical settings where ARM is not available since it may yield further information complementary to the DRE so as to guide therapy for CC patients [23].

ARM was available for 44.3% of GEs, but only 15% had access to high-resolution/high-definition ARM (which provides a continuous and dynamic spatiotemporal mapping of anorectal pressures compared to conventional ARM), enabling easier and more detailed data interpretation [38]. ARM + BET, as indicated by the Rome IV criteria, are important tools to diagnose functional defecation disorders, a frequent cause of refractory CC [6]. The scarce use of ARM and BET is probably the cause of many missed diagnoses of functional defecation disorders, leading patients to undergo ineffective therapeutic trials and frustrating both patients and GEs. Furthermore, radiopaque markers of colonic transit time were available in only about a half of centers. This simple test may suggest a delayed colonic transit time, guiding further therapies. The relatively scarce availability of anorectal physiology tests (e.g., ARM, Rx-defecography, balloon expulsion test, defeco-MRI) suggests, again, a low interest in appropriately managing CC despite its high prevalence. Furthermore, GEs in the northern and central areas of Italy have more frequent access to these diagnostic tests compared to GEs from the southern areas of Italy or the islands.

We acknowledge that having a dedicated outpatient clinic for DGBIs in every gastroenterology practice is in practice impossible, and the limited availability of different diagnostic tests for CC may limit the effective management of these patients. To try to overcome these problems, we are currently developing specific diagnostic–therapeutic pathways for CC patients (starting with primary care physicians and based on the results of this survey) in order to design a clear pathway to follow to optimize their management. In these diagnostic–therapeutic pathways, we will identify the role of the general practitioner in the management of these patients, and when a patient needs to be referred to tertiary centers for the management of troublesome or difficult CC patients. It is also important for each gastroenterology setting to know where to refer difficult and/or refractory CC patients. It would be desirable to identify referral centers in the various regions to which such patients could be referred to improve their management and appropriate referral.

Concerning the therapeutic approach to CC, diet and lifestyle advice are considered the “first-line” therapy, followed by fiber supplementation as a second-line therapy and macrogol as a third-line therapy. Clinical experience suggests that over-the-counter herbal products are usually among patients’ preferred treatments. Some patients consider these products safer compared with pharmacological therapies [39]. A common misconception among patients is that everything “natural” must be good or harmless, so they fail to consider the potential metabolic interactions between herbal remedies and the medications they are already taking, or the possible adverse events they can induce [40,41,42]. Conversely to patients’ perceptions and in line with international guidelines, GEs prefer suggesting therapies such as macrogol or fiber rather than herbal products as first-line therapies.

Prucalopride and linaclotide are not considered among the preferred therapeutic options. Their prescription is more frequent from the fifth to the twelfth therapeutic line. Enemas, suppositories and probiotics are preferred and generally prescribed before them. Prucalopride and linaclotide have been demonstrated to be effective and safe as second-line therapies for the treatment of CC [24], and prucalopride currently has the higher treatment persistence and adherence compared with other CC prescription medications in a United States-based study [43]. Despite current evidence, their prescription in Italy is limited. This is probably linked to a lack of awareness of these molecules (mechanism of action, dosages, etc.) on the part of many physicians and also their high monthly cost [4,44].

In this “ranking of prescription”, TAI and PFR are placed from the twelfth to the fifteenth position, but before surgical options and sacral neuromodulation, which are considered by GEs among the last therapeutic choices. In this regard, colectomy is frequently considered the last choice, and anorectal surgery is immediately before colectomy. Regarding TAI, there is emerging evidence which confirms its utility in the treatment of CC, at least in the short term [45]. This treatment, which in future can represent an alternative to surgery for refractory constipated patients, is more widely known and used in surgical settings compared to GE. This is demonstrated by the fact that the most recent evidence has come from studies carried out mainly by surgeons [45]. TAI can also be considered a bridge therapy for patients waiting to undergo physiology testing and who complain about severe refractory constipation symptoms [46]. This indication may be even more significant considering the low availability of such tests in Italy, as shown by the results of our survey. PFR was positioned at the same level as TAI among respondent GEs. This may be explained by clinical practice, where the waiting lists for PFR are usually very long in Italy, and patients often have to wait a considerable amount of time to start their course of PFR.

Our study has some limitations. Firstly, only about 17% of FISMAD participants responded to the survey, but it is likely that they were the GEs most involved with CC. Secondly, CC patients are also frequently managed by other specialists rather than GEs and by their general practitioners. However, the point of view on the management of chronic constipation by general practitioners was beyond the scope of our survey and had already been evaluated about ten years ago [47]. Moreover, one could possibly argue that the geographic distribution of the respondents may have affected the results, but our survey generally mirrors the distribution of GEs in Italy. Finally, the GEs were not asked to specify their precise affiliation, and this could have led some GEs working in the same structure/clinic to answer the survey, influencing the reliability of the results regarding some aspects of the survey (e.g., presence/absence of some diagnostic tools).

## 5. Conclusions

We think that the present survey gives an interesting picture of the everyday management of CC by Italian GEs. It identifies some critical aspects to improve (e.g., the scarce use of DRE and validated questionnaires in clinical practice and the lack of a widespread availability of anorectal physiology testing). Therefore, this could very well represent a first step to developing educational programs, including specialization courses, aimed at improving the management of this widespread condition. In this regard, specific diagnostic–therapeutic pathways for CC patients (starting with primary care physicians and based on the results of this survey) are needed.

## Figures and Tables

**Table 1:** Demographic features of respondents to the survey. Statistics: frequency (%) or median (IQR).

Demographic Feature	Value
Male/Female	117 (49.58%)/119 (50.42%)
Age	48 (26)
Geographic area	
Northern Italy	117 (49.57%)
Central Italy	40 (16.95%)
Southern Italy and islands	79 (33.47%)

**Table 2:** Diagnostic scores, questionnaire use and use of DRE in clinical practice for CC patients. Statistics: frequency (%).

	Use of Rome IV Criteria Questionnaire	Use of Bristol Stool Scale	Use of a Score to Assess Symptom Severity	Use of a Score to Assess Impact on QoL	DRE Always Performed
Overall routine utilization	177/236 (75.0%)	175/236 (74.1%)	91/236 (38.6%)	86/236 (36.4%)	134/236 (56.8%)
Age groups					
≤40 years (92)	65/92 (70.6%)	68/92 (73.9%)	22/92 (23.9%)	16/92 (17.4%)	29/92 (31.5%)
>40 years (144)	112/144 (77.9%)	107/144 (74.3%)	71/144 (49.3%)	70/144 (48.6%)	105/144 (72.9%)
*p*-value	0.218	0.849	<0.001	<0.001	<0.001
DGBI outpatient clinic					
Yes (99)	80/99 (80.8%)	81/99 (88.0%)	51/99 (51.5%)	44/99 (44.4%)	62/99 (62.6%)
No (137)	97/137 (70.8%)	94/137 (68.6%)	42/137 (30.7%)	42/137 (30.7%)	72/137 (52.5%)
*p*-value	0.08	0.022	0.001	0.03	0.139

**Table 3:** Diagnostic test availability for CC and its distribution in the different geographic areas in Italy. Statistics: frequency (%).

	Overall Availability (*n* = 236)	Northern Italy (*n* = 117)	Central Italy (*n* = 40)	Southern Italy and Islands (*n* = 79)	*p*-Value
Colonoscopy	231/236 (97.4%)	115/117 (98.3%)	39/40 (97.5%)	75/79 (94.9%)	0.902
CT–Colonoscopy	173/236 (73.3%)	95/117 (81.2%)	31/44 (77.5%)	47/79 (59.5%)	0.007
Conventional Anorectal Manometry (ARM)	97/236 (41.1%)	60/117 (51.3%)	11/40 (27.5%)	25/79 (31.6%)	0.004
High-resolution/high-definition ARM	36/236 (15.2%)	27/117 (23.1%)	4/40 (10.0%)	5/79 (6.3%)	0.005
Balloon expulsion test	45/236 (19.1%)	29/117 (24.8%)	7/40 (17.5%)	9/79 (11.4%)	0.046
Transanal ultrasound	124/236 (52.5%)	71/117 (60.7%)	27/40 (67.5%)	26/79 (32.9%)	<0.001
Dynamic Transperineal Ultrasound	34/236 (14.4%)	20/117 (17.1%)	8/40 (20.0%)	6/79 (7.6%)	0.111
Defeco-MRI	75/236 (31.8%)	42/117 (35.9%)	13/40 (32.5%)	20/79 (25.3%)	0.379
Perineography	36/236 (15.2%)	25/117 (21.4%)	4/40 (10.0%)	7/79 (8.9%)	0.043
Radiopaque Markers Colonic Transit Time	122/236 (51.7%)	70/117 (59.8%)	21/40 (52.5%)	31/79 (39.2%)	0.027
Colonic Transit Scintigraphy	29/236 (12.3%)	17/117 (14.5%)	6/40 (15.0%)	6/79 (7.6%)	0.35
Neurophysiologic Study of Pelvic Floor	33/236 (14.0%)	19/117 (16.2%)	8/40 (20.0%)	6/79 (7.6%)	0.127

## Data Availability

The data presented in this study are available upon request from the corresponding author (the data are not publicly available due to privacy or ethical restrictions).

## Supplementary Materials

The following supporting information can be downloaded at: https://www.mdpi.com/article/10.3390/jcm13206047/s1, Figure S1: Preferred therapies for chronic constipation among Italian GE. For each therapeutic preference (from 1 to 18), the frequencies of prescribed therapies are reported.
