# Supplementary material for: The Diagnosis and Management of Chronic Constipation in Italy: Results from a Survey Conducted among Italian Gastroenterologists
Source: J Clin Med. 2024 Oct 10;13(20):6047. doi: 10.3390/jcm13206047 (PMC11508448; doi:10.3390/jcm13206047)
Supplement: Supplementary file 1 [file jcm-13-06047-s001.zip › jcm-3249195-supplementary.pdf]

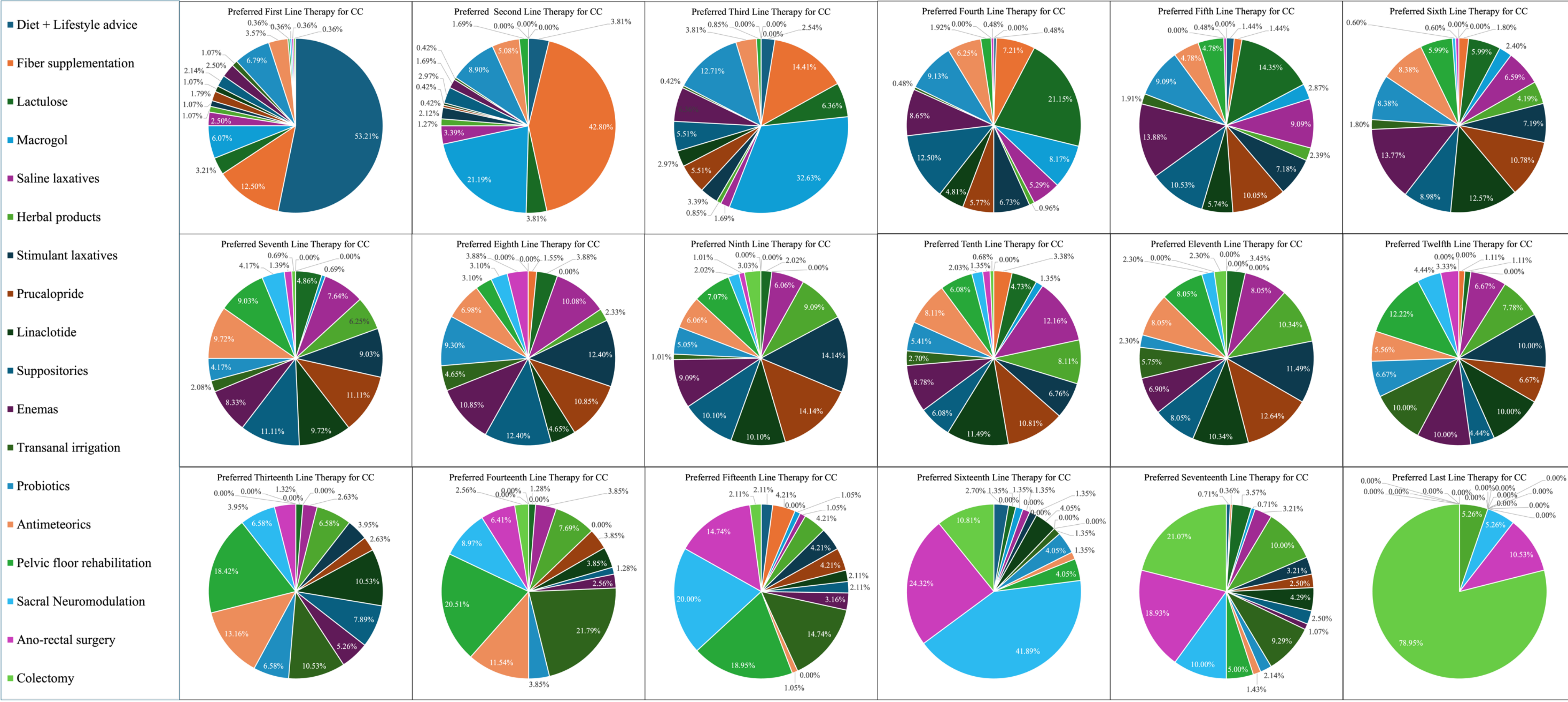

Figure S1. Preferred therapies for chronic constipation among Italian GE. For each therapeutic preference (from 1 to 18) the frequencies of prescribed therapies are reported
